# 3-Hy­droxy-2-(4-hy­droxy­phen­yl)-4*H*-chromen-4-one

**DOI:** 10.1107/S1600536810053407

**Published:** 2011-01-08

**Authors:** Michał Wera, Vasyl G. Pivovarenko, Jerzy Błażejowski

**Affiliations:** a Faculty of Chemistry, University of Gdańsk, J. Sobieskiego 18, 80-952 Gdańsk, Poland; b Faculty of Chemistry, Kyiv Taras Shevchenko National University, Volodymyrska 64, 01033 Kyiv, Ukraine

## Abstract

In the title compound, C_15_H_10_O_4_, the benzene ring is twisted at an angle of 20.7 (1)° relative to the 4*H*-chromene skeleton. In the crystal, adjacent mol­ecules are linked *via* a network of O—H⋯O and C—H⋯O hydrogen bonds. The mean planes of adjacent 4*H*-chromene moieties are parallel or oriented at an angle of 20.9 (1)° in the crystal structure.

## Related literature

For general background to the properties of flavones (deriva­tives of 2-phenyl-4*H*-chromen-4-one) and fluorescence of flavonols (derivatives of 3-hy­droxy-2-phenyl-4*H*-chromen-4-one), see: Bader *et al.* (2003 ▶); Choulier *et al.* (2010 ▶); Demchenko (2009 ▶); Klymchenko & Demchenko (2003 ▶); Nijveldt *et al.* (2001 ▶); Pivovarenko *et al.* (2004 ▶); Roshal *et al.* (2003 ▶); Sengupta & Kasha (1979 ▶). For related structures, see: Etter *et al.* (1986 ▶); Kumar *et al.* (1998 ▶); Waller *et al.* (2003 ▶). For inter­molecular inter­actions, see: Aakeröy *et al.* (1992 ▶); Novoa *et al.* (2006 ▶). For the synthesis, see: Bader *et al.* (2003 ▶); Sobottka *et al.* (2000 ▶).
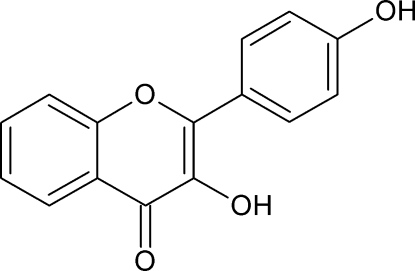


## Experimental

### 

#### Crystal data

C_15_H_10_O_4_
*M* *_r_* = 254.23
Monoclinic, 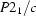

*a* = 3.7897 (3) Å
*b* = 17.6380 (15) Å
*c* = 16.7745 (16) Å
β = 90.968 (9)°
*V* = 1121.09 (17) Å^3^
*Z* = 4
Mo *K*α radiation
μ = 0.11 mm^−1^
*T* = 295 K
0.6 × 0.2 × 0.2 mm

#### Data collection

Oxford Diffraction Gemini R Ultra Ruby CCD diffractometer
Absorption correction: multi-scan (*CrysAlis RED*; Oxford Diffraction, 2008 ▶) *T*_min_ = 0.329, *T*_max_ = 1.000
9273 measured reflections
1979 independent reflections
920 reflections with *I* > 2σ(*I*)
*R*_int_ = 0.075

#### Refinement

*R*[*F* ^2^ > 2σ(*F* ^2^)] = 0.060
*wR*(*F* ^2^) = 0.155
*S* = 0.84
1979 reflections
179 parameters
H atoms treated by a mixture of independent and constrained refinement
Δρ_max_ = 0.28 e Å^−3^
Δρ_min_ = −0.31 e Å^−3^

### 

Data collection: *CrysAlis CCD* (Oxford Diffraction, 2008 ▶); cell refinement: *CrysAlis RED* (Oxford Diffraction, 2008 ▶); data reduction: *CrysAlis RED*; program(s) used to solve structure: *SHELXS97* (Sheldrick, 2008 ▶); program(s) used to refine structure: *SHELXL97* (Sheldrick, 2008 ▶); molecular graphics: *ORTEP-3* (Farrugia, 1997 ▶); software used to prepare material for publication: *SHELXL97* and *PLATON* (Spek, 2009 ▶).

## Supplementary Material

Crystal structure: contains datablocks global, I. DOI: 10.1107/S1600536810053407/xu5114sup1.cif

Structure factors: contains datablocks I. DOI: 10.1107/S1600536810053407/xu5114Isup2.hkl

Additional supplementary materials:  crystallographic information; 3D view; checkCIF report

## Figures and Tables

**Table 1:** Hydrogen-bond geometry (Å, °)

*D*—H⋯*A*	*D*—H	H⋯*A*	*D*⋯*A*	*D*—H⋯*A*
O11—H11⋯O19^i^	0.83 (5)	2.10 (5)	2.832 (4)	148 (4)
O19—H19⋯O12^ii^	0.91 (5)	1.79 (5)	2.705 (4)	176 (5)
C7—H7⋯O11^iii^	0.93	2.47	3.267 (4)	144

Symmetry codes: (i) 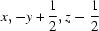; (ii) 
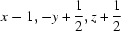
; (iii) 
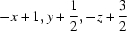
.

**Fig. 1.**
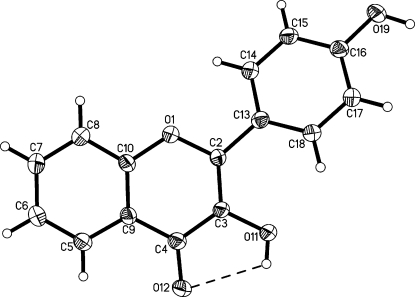
The molecular structure of the title compound showing the atom labeling scheme. Displacement ellipsoids are drawn at the 25% probability level, and H atoms are shown as small spheres of arbitrary radius. The O—H···O hydrogen bond is indicated by a dashed line.

**Fig. 2.**
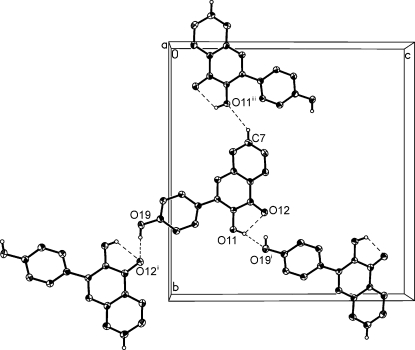
The arrangement of the molecules in the crystal structure. The O–H···O and C–H···O hydrogen bonds are represented by dashed lines. H atoms not involved in interactions have been omitted. [Symmetry codes: (i) x, –y + 1/2, z – 1/2; (ii) x – 1, –y + 1/2, z + 1/2; (iii) –x + 1, y + 1/2, –z + 3/2.]

## supplementary crystallographic information

### Comment

Flavones (derivatives of 2-phenyl-4*H*-chromen-4-one) appear in numerous natural systems and have been comprehensively investigated in view of their antioxidant features (Nijveldt *et al.*, 2001). Related to flavones, 3-hydroxy-2-phenyl-4*H*-chromen-4-one (flavonols) exhibit dual fluorescence in the condensed phases resulting from the Excited State Intramolecular Proton Transfer (ESIPT) (Sengupta & Kasha, 1979). In flavonols this phenomenon is strongly affected by molecules from their environment, which makes the compounds interesting fluorescent sensors for analytical applications in chemistry, biology, biochemistry, ecology and medicine (Klymchenko & Demchenko, 2003; Demchenko, 2009; Choulier *et al.*, 2010). Continuing our investigations into the physical chemistry of flavonols (Bader *et al.*, 2003; Roshal *et al.*, 2003; Pivovarenko *et al.*, 2004), we present the crystal structure of 3-hydroxy-2-(4-hydroxyphenyl)-4*H*-chromen-4-one in the hope that its structural and fluorescent features will appear interesting and helpful in its practical applications.

In the title compound (Fig. 1), the bond lengths and angles characterizing the geometry of the 2-phenyl-4*H*-chromen-4-one moiety are typical of this group of compounds (Etter *et al.*, 1986; Kumar *et al.*, 1998; Waller *et al.*, 2003). With respective average deviations from planarity of 0.0187 (1)° and 0.0041 (1)°, the 4*H*-chromene and benzene ring systems are oriented at a dihedral angle of 20.7 (1)° (in the case of flavonol this angle is 5.5 (1)° (Etter *et al.*, 1986)). The mean planes of the adjacent 4*H*-chromen-4-one moieties are either parallel (remain at an angle of 0.0 (1)°) or inclined at 20.9 (1)°.

The crystal structure of the title compound is stabilized by a network of O—H···O (Aakeröy *et al.*, 1992) (Table 1, Fig. 2) and C—H···O (Novoa *et al.*, 2006) (Table 1, Fig. 2) hydrogen bonds, and by non-specific dispersive interactions. Each of the two OH groups is involved in hydrogen bonds as H atom acceptor and donor. The O11—H11···O12 intramolecular hydrogen bond (Table 1, Figs. 1 and 2) is the one involved in the ESIPT phenomenon characteristic of flavonols (Sengupta & Kasha, 1979).

### Experimental

The title compound was synthesized in two steps. First, 3-hydroxy-2-(4-methoxyphenyl)-4*H*-chromen-4-one was prepared by alkaline condensation of 4-methoxybenzaldehyde with 1-(2-hydroxyphenyl)ethanone and subsequent oxidative heterocyclization of the product with hydrogen peroxide (the light green-yellow precipitate of the product was recrystallized twice from a 1% solution of acetic acid in ethanol) (Bader *et al.*, 2003). Next, the 3-hydroxy-2-(4-methoxyphenyl)-4*H*-chromen-4-one thus obtained was converted to 3-hydroxy-2-(4-hydroxyphenyl)-4*H*-chromen-4-one by maintaining a solution of the former compound in molten pyridinium chloride at 495 K for 20 minutes, then cooling the reactant mixture, and pouring it into 1% aqueous HCl. Pale brown crystals suitable for X-ray investigations were grown from DMF solutions of the filtered precipitate of the final product (m.p. = 557–558 K; lit. 555–558 K (Sobottka *et al.*, 2000)).

### Refinement

H atoms of C—H bonds were positioned geometrically with H = 0.93 Å and constrained to ride on their parent atoms with *U*_iso_(H) = 1.2*U*_eq_(C). H atoms involved in O—H···O hydrogen bonds were located on a difference Fourier map and refined isotropically with *U*_iso_(H) = 1.5*U*_eq_(O).

### Crystal data

**Table:**

C_15_H_10_O_4_	*F*(000) = 528
*M**_r_* = 254.23	*D*_x_ = 1.506 Mg m^−^^3^
Monoclinic, *P*2_1_/*c*	Mo *K*α radiation, λ = 0.71073 Å
Hall symbol: -P 2ybc	Cell parameters from 1979 reflections
*a* = 3.7897 (3) Å	θ = 3.4–25.0°
*b* = 17.6380 (15) Å	µ = 0.11 mm^−^^1^
*c* = 16.7745 (16) Å	*T* = 295 K
β = 90.968 (9)°	Needle, pale brown
*V* = 1121.09 (17) Å^3^	0.6 × 0.2 × 0.2 mm
*Z* = 4	

### Data collection

**Table:**

Oxford Diffraction Gemini R Ultra Ruby CCD diffractometer	1979 independent reflections
Radiation source: Enhance (Mo) X-ray Source	920 reflections with *I* > 2σ(*I*)
graphite	*R*_int_ = 0.075
Detector resolution: 10.4002 pixels mm^-1^	θ_max_ = 25.0°, θ_min_ = 3.4°
ω scans	*h* = −4→4
Absorption correction: multi-scan (*CrysAlis RED*; Oxford Diffraction, 2008)	*k* = −20→20
*T*_min_ = 0.329, *T*_max_ = 1.000	*l* = −19→19
9273 measured reflections	

### Refinement

**Table:**

Refinement on *F*^2^	Secondary atom site location: difference Fourier map
Least-squares matrix: full	Hydrogen site location: inferred from neighbouring sites
*R*[*F*^2^ > 2σ(*F*^2^)] = 0.060	H atoms treated by a mixture of independent and constrained refinement
*wR*(*F*^2^) = 0.155	*w* = 1/[σ^2^(*F*_o_^2^) + (0.0893*P*)^2^] where *P* = (*F*_o_^2^ + 2*F*_c_^2^)/3
*S* = 0.84	(Δ/σ)_max_ < 0.001
1979 reflections	Δρ_max_ = 0.28 e Å^−^^3^
179 parameters	Δρ_min_ = −0.31 e Å^−^^3^
0 restraints	Extinction correction: *SHELXL97* (Sheldrick, 2008), Fc^*^=kFc[1+0.001xFc^2^λ^3^/sin(2θ)]^-1/4^
Primary atom site location: structure-invariant direct methods	Extinction coefficient: 0.011 (3)

### Special details

**Table:**

Geometry. All e.s.d.'s (except the e.s.d. in the dihedral angle between two l.s. planes) are estimated using the full covariance matrix. The cell e.s.d.'s are taken into account individually in the estimation of e.s.d.'s in distances, angles and torsion angles; correlations between e.s.d.'s in cell parameters are only used when they are defined by crystal symmetry. An approximate (isotropic) treatment of cell e.s.d.'s is used for estimating e.s.d.'s involving l.s. planes.
Refinement. Refinement of *F*^2^ against ALL reflections. The weighted *R*-factor *wR* and goodness of fit *S* are based on *F*^2^, conventional *R*-factors *R* are based on *F*, with *F* set to zero for negative *F*^2^. The threshold expression of *F*^2^ > σ(*F*^2^) is used only for calculating *R*-factors(gt) *etc*. and is not relevant to the choice of reflections for refinement. *R*-factors based on *F*^2^ are statistically about twice as large as those based on *F*, and *R*- factors based on ALL data will be even larger.

### Fractional atomic coordinates and isotropic or equivalent isotropic displacement parameters (Å^2^)

**Table:**

	*x*	*y*	*z*	*U*_iso_*/*U*_eq_	
O1	0.2942 (6)	0.46752 (12)	0.80136 (13)	0.0490 (7)	
C2	0.2180 (8)	0.39182 (18)	0.8026 (2)	0.0416 (9)	
C3	0.2703 (9)	0.34891 (18)	0.7361 (2)	0.0430 (9)	
C4	0.4184 (9)	0.3797 (2)	0.6655 (2)	0.0470 (9)	
C5	0.6344 (8)	0.4972 (2)	0.6004 (2)	0.0507 (10)	
H5	0.6885	0.4702	0.5546	0.061*	
C6	0.6924 (9)	0.5739 (2)	0.6035 (2)	0.0570 (10)	
H6	0.7830	0.5990	0.5595	0.068*	
C7	0.6161 (10)	0.6139 (2)	0.6719 (2)	0.0602 (11)	
H7	0.6540	0.6660	0.6732	0.072*	
C8	0.4854 (9)	0.5782 (2)	0.7379 (2)	0.0538 (10)	
H8	0.4373	0.6054	0.7839	0.065*	
C9	0.4944 (8)	0.45958 (18)	0.66600 (19)	0.0423 (9)	
C10	0.4267 (8)	0.50063 (19)	0.7343 (2)	0.0431 (9)	
O11	0.1984 (8)	0.27345 (14)	0.73772 (16)	0.0663 (9)	
H11	0.167 (12)	0.257 (3)	0.692 (3)	0.099*	
O12	0.4757 (7)	0.33773 (14)	0.60629 (15)	0.0662 (8)	
C13	0.0981 (8)	0.36660 (19)	0.8811 (2)	0.0418 (9)	
C14	0.1704 (9)	0.4109 (2)	0.9483 (2)	0.0488 (9)	
H14	0.2816	0.4575	0.9424	0.059*	
C15	0.0792 (9)	0.3865 (2)	1.0232 (2)	0.0514 (10)	
H15	0.1291	0.4166	1.0675	0.062*	
C16	−0.0861 (9)	0.3175 (2)	1.0325 (2)	0.0479 (9)	
C17	−0.1637 (9)	0.2732 (2)	0.9672 (2)	0.0502 (10)	
H17	−0.2771	0.2269	0.9735	0.060*	
C18	−0.0727 (8)	0.29790 (19)	0.8922 (2)	0.0463 (9)	
H18	−0.1270	0.2678	0.8481	0.056*	
O19	−0.1701 (7)	0.29545 (15)	1.10853 (15)	0.0614 (8)	
H19	−0.285 (11)	0.250 (3)	1.110 (3)	0.092*	

### Atomic displacement parameters (Å^2^)

**Table:**

	*U*^11^	*U*^22^	*U*^33^	*U*^12^	*U*^13^	*U*^23^
O1	0.0679 (16)	0.0378 (15)	0.0414 (16)	−0.0017 (11)	0.0061 (12)	0.0001 (11)
C2	0.053 (2)	0.036 (2)	0.036 (2)	−0.0001 (15)	−0.0006 (16)	0.0032 (16)
C3	0.061 (2)	0.033 (2)	0.035 (2)	0.0025 (16)	−0.0020 (17)	0.0000 (16)
C4	0.057 (2)	0.045 (2)	0.039 (2)	0.0033 (17)	−0.0027 (17)	−0.0001 (17)
C5	0.061 (2)	0.051 (3)	0.040 (2)	0.0038 (18)	0.0021 (18)	0.0014 (17)
C6	0.067 (2)	0.055 (3)	0.049 (3)	−0.003 (2)	0.0049 (19)	0.0112 (19)
C7	0.080 (3)	0.044 (2)	0.057 (3)	−0.007 (2)	0.002 (2)	0.007 (2)
C8	0.068 (2)	0.043 (2)	0.051 (3)	−0.0048 (19)	0.0036 (19)	−0.0028 (18)
C9	0.049 (2)	0.040 (2)	0.038 (2)	0.0041 (16)	0.0008 (17)	0.0045 (16)
C10	0.048 (2)	0.044 (2)	0.036 (2)	0.0011 (17)	0.0008 (17)	0.0049 (16)
O11	0.122 (2)	0.0371 (16)	0.0400 (17)	−0.0105 (14)	0.0089 (16)	−0.0016 (12)
O12	0.111 (2)	0.0486 (16)	0.0399 (17)	0.0058 (14)	0.0140 (14)	−0.0030 (13)
C13	0.048 (2)	0.037 (2)	0.040 (2)	0.0051 (16)	−0.0046 (16)	−0.0003 (15)
C14	0.061 (2)	0.040 (2)	0.045 (2)	−0.0006 (17)	0.0034 (18)	0.0014 (18)
C15	0.071 (3)	0.044 (2)	0.039 (2)	0.0022 (18)	0.0014 (18)	−0.0035 (17)
C16	0.057 (2)	0.044 (2)	0.043 (2)	0.0084 (18)	0.0049 (17)	0.0009 (17)
C17	0.061 (2)	0.042 (2)	0.047 (3)	−0.0046 (17)	0.0065 (18)	0.0012 (17)
C18	0.056 (2)	0.042 (2)	0.042 (2)	−0.0007 (16)	0.0041 (17)	−0.0033 (16)
O19	0.091 (2)	0.0553 (18)	0.0380 (17)	−0.0007 (14)	0.0134 (14)	0.0056 (13)

### Geometric parameters (Å, °)

**Table:**

O1—C2	1.366 (4)	C8—H8	0.9300
O1—C10	1.370 (4)	C9—C10	1.383 (5)
C2—C3	1.365 (4)	O11—H11	0.82 (5)
C2—C13	1.469 (4)	C13—C18	1.388 (4)
C3—O11	1.359 (4)	C13—C14	1.394 (5)
C3—C4	1.427 (5)	C14—C15	1.378 (5)
C4—O12	1.261 (4)	C14—H14	0.9300
C4—C9	1.438 (5)	C15—C16	1.379 (5)
C5—C6	1.372 (5)	C15—H15	0.9300
C5—C9	1.397 (5)	C16—C17	1.373 (5)
C5—H5	0.9300	C16—O19	1.375 (4)
C6—C7	1.381 (5)	C17—C18	1.381 (5)
C6—H6	0.9300	C17—H17	0.9300
C7—C8	1.374 (5)	C18—H18	0.9300
C7—H7	0.9300	O19—H19	0.91 (4)
C8—C10	1.387 (5)		
			
C2—O1—C10	120.6 (3)	C5—C9—C4	122.7 (3)
O1—C2—C3	119.7 (3)	O1—C10—C9	122.2 (3)
O1—C2—C13	112.2 (3)	O1—C10—C8	116.5 (3)
C3—C2—C13	128.0 (3)	C9—C10—C8	121.3 (3)
O11—C3—C2	119.7 (3)	C3—O11—H11	110 (3)
O11—C3—C4	118.1 (3)	C18—C13—C14	117.8 (3)
C2—C3—C4	122.1 (3)	C18—C13—C2	122.4 (3)
O12—C4—C3	120.4 (3)	C14—C13—C2	119.7 (3)
O12—C4—C9	122.8 (3)	C15—C14—C13	120.9 (3)
C3—C4—C9	116.7 (3)	C15—C14—H14	119.6
C6—C5—C9	120.1 (3)	C13—C14—H14	119.6
C6—C5—H5	119.9	C14—C15—C16	120.0 (3)
C9—C5—H5	119.9	C14—C15—H15	120.0
C5—C6—C7	119.9 (4)	C16—C15—H15	120.0
C5—C6—H6	120.0	C17—C16—O19	122.0 (3)
C7—C6—H6	120.0	C17—C16—C15	120.2 (3)
C8—C7—C6	121.3 (4)	O19—C16—C15	117.8 (3)
C8—C7—H7	119.4	C16—C17—C18	119.6 (3)
C6—C7—H7	119.4	C16—C17—H17	120.2
C7—C8—C10	118.5 (4)	C18—C17—H17	120.2
C7—C8—H8	120.8	C17—C18—C13	121.4 (3)
C10—C8—H8	120.8	C17—C18—H18	119.3
C10—C9—C5	118.8 (3)	C13—C18—H18	119.3
C10—C9—C4	118.5 (3)	C16—O19—H19	113 (3)
			
C10—O1—C2—C3	−0.8 (4)	C5—C9—C10—O1	−179.1 (3)
C10—O1—C2—C13	176.8 (3)	C4—C9—C10—O1	0.8 (5)
O1—C2—C3—O11	179.2 (3)	C5—C9—C10—C8	1.9 (5)
C13—C2—C3—O11	2.1 (5)	C4—C9—C10—C8	−178.3 (3)
O1—C2—C3—C4	2.9 (5)	C7—C8—C10—O1	−179.6 (3)
C13—C2—C3—C4	−174.2 (3)	C7—C8—C10—C9	−0.5 (5)
O11—C3—C4—O12	0.9 (5)	O1—C2—C13—C18	164.2 (3)
C2—C3—C4—O12	177.2 (3)	C3—C2—C13—C18	−18.5 (5)
O11—C3—C4—C9	−179.4 (3)	O1—C2—C13—C14	−18.9 (4)
C2—C3—C4—C9	−3.1 (5)	C3—C2—C13—C14	158.5 (3)
C9—C5—C6—C7	0.8 (5)	C18—C13—C14—C15	0.9 (5)
C5—C6—C7—C8	0.6 (6)	C2—C13—C14—C15	−176.2 (3)
C6—C7—C8—C10	−0.8 (6)	C13—C14—C15—C16	0.0 (5)
C6—C5—C9—C10	−2.0 (5)	C14—C15—C16—C17	−0.7 (5)
C6—C5—C9—C4	178.2 (3)	C14—C15—C16—O19	179.7 (3)
O12—C4—C9—C10	−179.1 (3)	O19—C16—C17—C18	−179.8 (3)
C3—C4—C9—C10	1.2 (4)	C15—C16—C17—C18	0.6 (5)
O12—C4—C9—C5	0.7 (5)	C16—C17—C18—C13	0.3 (5)
C3—C4—C9—C5	−178.9 (3)	C14—C13—C18—C17	−1.0 (5)
C2—O1—C10—C9	−1.1 (4)	C2—C13—C18—C17	176.0 (3)
C2—O1—C10—C8	178.0 (3)		

### Hydrogen-bond geometry (Å, °)

**Table:**

*D*—H···*A*	*D*—H	H···*A*	*D*···*A*	*D*—H···*A*
O11—H11···O12	0.83 (5)	2.35 (5)	2.707 (4)	107 (4)
O11—H11···O19^i^	0.83 (5)	2.10 (5)	2.832 (4)	148 (4)
O19—H19···O12^ii^	0.91 (5)	1.79 (5)	2.705 (4)	176 (5)
C7—H7···O11^iii^	0.93	2.47	3.267 (4)	144

Symmetry codes:  (i) *x*, −*y*+1/2, *z*−1/2; (ii) *x*−1, −*y*+1/2, *z*+1/2; (iii) −*x*+1, *y*+1/2, −*z*+3/2.
